# Future development of biologically relevant dosimetry

**DOI:** 10.1259/bjr.20140392

**Published:** 2014-11-20

**Authors:** H Palmans, H Rabus, A L Belchior, M U Bug, S Galer, U Giesen, G Gonon, G Gruel, G Hilgers, D Moro, H Nettelbeck, M Pinto, A Pola, S Pszona, G Schettino, P H G Sharpe, P Teles, C Villagrasa, J J Wilkens

**Affiliations:** ^1^ Acoustics and Ionising Radiation Division, National Physical Laboratory (NPL), Teddington, Middlesex, UK; ^2^ Medical Physics, EBG MedAustron GmbH, Wiener Neustadt, Austria; ^3^ Abteilung Ionisierende Strahlung, Physikalisch-Technische Bundesanstalt (PTB), Braunschweig, Germany; ^4^ Associação do Instituto Superior Técnico para a Investigação e Desenvolvimento (IST-ID), Lisboa, Portugal; ^5^ Unité Radioprotection de I'Homme, Institut de Radioprotection et de Sûreté Nucléaire (IRSN), Fontenay-aux-Roses, France; ^6^ Laboratori Nazionale di Legnaro, Istituto Nazionale di Fisica Nucleare, Padova, Italy; ^7^ Centro Ricerche Casaccia, Istituto Nazionale di Metrologia delle Radiazioni Ionizzanti ENEA-INMRI, Rome, Italy; ^8^ Department of Energy, Politecnico di Milano, Milan, Italy; ^9^ Kierownik Zatladu Interdyscyplinarnych Zastosowañ Fizyki, Narodowe Centrum Badán Jądrowych, Otwock-Swierk, Poland; ^10^ Unidade de Protecção e Segurança Radiológica, Instituto Tecnológico e Nuclear, Instituto Superior Técnico, Sacavém, Portugal; ^11^ Department of Radiation Oncology, Technische Universität München, Klinikum rechts der Isar, Munich, Germany

## Abstract

Proton and ion beams are radiotherapy modalities of increasing importance and interest. Because of the different biological dose response of these radiations as compared with high-energy photon beams, the current approach of treatment prescription is based on the product of the absorbed dose to water and a biological weighting factor, but this is found to be insufficient for providing a generic method to quantify the biological outcome of radiation. It is therefore suggested to define new dosimetric quantities that allow a transparent separation of the physical processes from the biological ones. Given the complexity of the initiation and occurrence of biological processes on various time and length scales, and given that neither microdosimetry nor nanodosimetry on their own can fully describe the biological effects as a function of the distribution of energy deposition or ionization, a multiscale approach is needed to lay the foundation for the aforementioned new physical quantities relating track structure to relative biological effectiveness in proton and ion beam therapy. This article reviews the state-of-the-art microdosimetry, nanodosimetry, track structure simulations, quantification of reactive species, reference radiobiological data, cross-section data and multiscale models of biological response in the context of realizing the new quantities. It also introduces the European metrology project, Biologically Weighted Quantities in Radiotherapy, which aims to investigate the feasibility of establishing a multiscale model as the basis of the new quantities. A tentative generic expression of how the weighting of physical quantities at different length scales could be carried out is presented.

The increased incidence of cancer as a leading cause of mortality worldwide^1,2^ has heightened the demand for radiotherapy and interest to develop advanced radiotherapy modalities particularly suited for the treatment of aggressive tumours. The clinical interest in high-energy protons or heavier ions (*e.g.* carbon ions) as a promising alternative to state-of-the-art megavoltage X-ray external beam therapy has therefore risen significantly within the last decade.^3–5^ Compared with megavoltage X-rays, particle beams can deliver similar dose distributions to the target volume whilst sparing normal healthy tissue. Since its inception in the 1950s and 1960s, >100,000 patients have been treated worldwide with particle beam therapy (approximately 85% with protons and approximately 15% with heavier ions, mainly carbon).^6^ At present, 43 proton and 8 carbon ion therapy facilities are in operation worldwide.^7^ An additional 25 proton and 3 carbon ion therapy centres are scheduled to commence operation by 2015^8^ and another 13 are in the planning stage.^9^ The global number of treatments performed per year with these therapies has risen from about 3000 patients in 2005 to about 14,000 patients in 2013.^6^

For the more common external radiotherapy modalities using high-energy photon and electron beams, it is adequate to quantify the administered amount of radiation by the quantity of absorbed dose to water, since tumour control probability and healthy tissue toxicity are observed to be independent of the radiation quality for these modalities. A standard relative measurement uncertainty of <2.5% is required for absorbed dose to water in the tumour,^10^ where primary measurement standards of this quantity can achieve a standard uncertainty as low as 0.3% for photon beams.^11^

For proton and carbon ion beams, however, the same biological effect occurs for a different value of absorbed dose when compared with that for high-energy photons. The current practice is that the dose administered in ion beam radiotherapy is quantified in terms of the photon-isoeffective dose, which is the product of the absorbed dose to water in the ion beam and a biological weighting factor [relative biological effectiveness (RBE)].^12^ The latter depends on both physical factors and biological processes. The physical component is related to the microscopic particle track structure, which depends on the particle type and energy. Its change with decreasing particle energy is believed to account for most of the variation of RBE along the ion track. The biological component is dependent on how cells respond to the distribution of energy deposition and ionization at various geometrical and temporal scales.

The issue of biological weighting factors in radiotherapy has been the subject of a recent joint report of the International Atomic Energy Agency (IAEA) and the International Commission on Radiation Units and Measurements (ICRU). In the report entitled “Relative biological effectiveness in ion beam therapy”,^13^ IAEA and ICRU point out that the growing use of radiotherapy modalities whose biological effect differs from that of high-energy photon beams heightens the need for consistency across the different radiotherapy modalities. They expressed their concern about a diversity of methods being used in radiotherapy to derive biological weighting factors, which vary for different modalities and are applied “in an often inconsistent manner leading to confusion in interpretation and possible risk to patients”.^13^ In order to reduce confusion and to aid in the comparison of treatment efficacies for different radiotherapy techniques, IAEA and ICRU propose a “universally agreed approach for the use of weighting factors … (to) …facilitate exchange of information and to improve collaboration between centres and within the radiation oncology community”.^13^

This clearly illustrates that the present approach is insufficient for providing a generic method to quantify the biological outcome of radiation dose, and, consequently, all centres have no other choice than to perform expensive biological characterizations of their beams. It is therefore desirable to establish new dosimetric quantities that allow a transparent separation of the physical processes from the biological ones. The Consultative Committee of Ionising Radiation (of the International Committee of Measures and Weights) has expressed strong support for defining such a new quantity, particularly for treatments involving the use of one or more multiplying factors to describe the corresponding biological effects of the absorbed dose.^14^

Microdosimetry and nanodosimetry have been developed to quantify the physical part of the radiation weighting factors, such as the transfer of energy to the cell and nucleus, the resulting radiation chemistry and the density of ionization in chromosomes or in and around the DNA. Monte Carlo simulations of lineal energy and ionization cluster distribution have been developed to aid these experimental methods. However, neither microdosimetry nor nanodosimetry on its own can fully describe the biological effects as a function of the distribution of energy deposition or ionization. In addition, present day methods measure ionization in gases or semiconductors so that these measurements are not necessarily representative of the energy deposition in tissue. Given the complexity of the initiation and occurrence of biological processes on various scales that depend on both ionization and non-ionization events (such as excitations and even local heating), a multiscale approach is needed to lay the foundation for new physical quantities relating track structure to RBE in proton and ion beam therapy.

This article reviews the state-of-the-art microdosimetry, nanodosimetry, track structure simulations, quantification of reactive species, reference radiobiological data, cross section data and multiscale models of biological response in the context of realizing the biologically relevant quantities suggested above. This is followed by a discussion on what are the relevant quantities that need to be measured and the prospect of new standardized biologically relevant dosimetric quantities. The progress of a European metrology project, Biologically Weighted Quantities in Radiotherapy (BioQuaRT),^15^ that initiated an investigation into the feasibility of the new quantities is also presented.

## STATE OF THE ART AND PROSPECTS OF UNDERPINNING TECHNOLOGIES

### Microdosimetry

Microdosimetry is the study and quantification of the spatial and temporal distribution of the interaction of radiation with sensitive volumes of matter at micrometre length scales.^16,17^ Regional microdosimetry studies the fluctuations of energy deposition in a micrometre-sized target without considering the spatial distribution of energy deposition within this target. Microdosimetric quantities are stochastic and therefore given in terms of particle (or event) interaction probabilities. The outcomes are usually considered as constituting part of the radiation quality of the investigated beam. The radiation field quality is meant as a physical measurable quantity, which is significant for primary effects on a biological system. Microdosimetry is concerned with ionizations, since these interactions are well correlated with damage to DNA, as demonstrated by Brenner and Ward.^18,19^

Relevant quantities in microdosimetry are:^17^

• *y*: the lineal energy, which is defined as the energy imparted to matter in the microscopic volume by a single event divided by the mean chord length in that volume
• *f*(*y*): the probability distribution of lineal energy
• 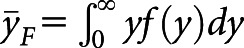: the first moment of *f*(*y*), also called the frequency mean lineal energy^20^
• 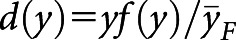: the dose distribution, which is important for obtaining the dose components of the microdosimetric spectrum. This distribution multiplied with a weighting function can be used to estimate the RBE of a beam^21^
• 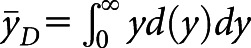: the first moment of *d*(*y*), also called the dose mean lineal energy.

The ordinary representation of a microdosimetric spectrum is a semi-logarithmic plot with the lineal energy, *y*, on the horizontal axis and the product *y* *d*(*y*) on the vertical axis. In this way, the area under the curve represents the contribution to the absorbed dose from the different lineal energy values. While the use of a tissue-equivalent medium as reference for microdosimetric spectra is widely accepted, it remains an unsolved question whether this is the most relevant medium for proposing a biologically relevant dosimetric quantity for radiotherapy. The requirement of atomic tissue equivalence stems from the microdosimetry of neutron fields where the local energy deposition is dominated by the low-energy secondary protons produced in nuclear interactions.^22^ If the formation of radical species in water was the only mechanism correlating microscopic energy depositions with DNA damage, then energy deposition, and thus lineal energy distributions, in water would be more relevant. Depending on the range of charged particles set in motion in the cellular and tissue environment, there may be need for a more sophisticated approach that considers the generation of secondary particles in a relevant target (*e.g.* nucleus, cytoplasm, cell or tissue matrix) as well as evaluating lineal energy in water.^23^ It can also not be ruled out that micrometric distributions of ionization are more relevant than lineal energy distributions, and it has, for example, been demonstrated experimentally that the same ionization distributions can be realized in different gas mixtures in a tissue-equivalent proportional counter (TEPC)^24^ from which it could be inferred that they are the same for a corresponding volume of water. Only further development of measurement capabilities to determine microdosimetric distributions of both ionization and lineal energy will enable definite conclusions to be drawn.

Microdosimetric spectra are commonly measured with gas-filled proportional counters.^25^ The electric field between the anode wire and the conductive wall of the counter makes electrons produced in gas by the radiation accelerate along field lines causing a charge avalanche. The amount of charge collected is proportional to the number of electron-ion pairs produced, and if the mean energy required to produce an electron-ion pair in the gas, *W*_gas_, is constant, the amount of charge collected is also proportional to the energy deposited within the counter volume.

**Figure 1.**
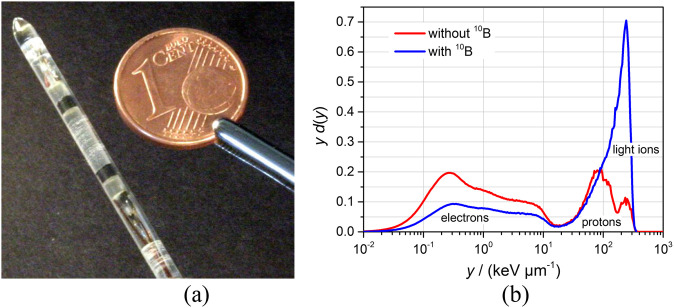
Photo of a twin-tissue-equivalent proportional counter dummy (a) and collected microdosimetric spectra in a thermal neutron beam for boron neutron capture therapy (BNCT) applications (b; data from Moro et al^28^). The only difference between the dummy and real detector is that the external sleeve is made of polymethyl methacrylate (PMMA) instead of aluminium. The simulated microdosimetric spectra were obtained for a 1-µm-sized site at the TAPIRO thermal column of ente per le nuove tecnologie, I'energia e l'ambiente (ENEA) (RomeItaly).

The most common proportional counters are large volume chambers operated at low pressure in order to measure ionizations in a macroscopic volume instead of a microscopic one.^16^ In these devices, various effects need to be corrected for as they contribute substantially to the measurement uncertainty.^17,25^ For example, the δ-ray effect, the V-effect and the scatter effect. All of which concern conditions where two charged particles traverse the large cavity, whereas in real tissue, only one of these would enter the small volume of interest. Another effect is the re-entry effect, which concerns a particle that has traversed the cavity and re-enters the detector volume, whereas in real tissue, it would miss the small volume of interest. Another issue is the need for calibration, which is usually carried out using sources of known characteristics or edges of maximum energy deposition in the lineal energy spectrum.^26,27^ A more superior device operating under similar principles is the mini-TEPC (Figure 1a), which has a much smaller volume than conventional TEPCs. This reduces the magnitude of various correction factors^29,30^ and also allows for the gas pressure to be much higher, which has led to its success for measuring lineal energy spectra in proton beams and neutron fields used in boron neutron capture therapy,^28,31^ the latter is shown in Figure 1b.

As mentioned above, microdosimetry performed by using a TEPC filled with low-density tissue-equivalent (TE) gas simulates site sizes in the range of the diameter of a cell nucleus. Two sites, for example, two spheres of different dimensions, one of tissue and one filled with gas, are said to be equivalent when the mean imparted energies are equal, that is, 
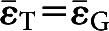
, where the subscript “T” stands for “tissue” and “G” for “gas”. The same equation can be written as: 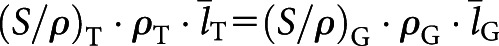, where *S/ρ* are the mass stopping powers, *ρ* the mass densities and the mean chord lengths of the sites. If the gas has the same atomic composition of the tissue, that is, 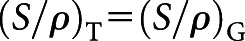, then 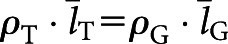. The last equation is used in experimental microdosimetry for properly adjusting the gas pressure when filling the TEPC with TE gas mixtures.^24^ In particular, it has been shown that the equivalence remains valid also for pure propane using a simple scaling factor.^24^

Silicon devices are being studied as microdosimeters since they can provide sensitive volumes that correspond to realistic microscopic sizes of the biological components of interest such as the cell, cell nucleus or chromosomes. Since silicon is not a tissue, the measurements performed through these detectors must be corrected for tissue inequivalence. Nevertheless, the simplicity, compactness, low cost, transportability, low power consumption and a low sensitivity to vibrations make silicon devices very promising for an easier approach to experimental microdosimetry.

The study of semiconductor detectors for microdosimetry dates back to 1980.^32^ Several devices (mainly diodes) have been employed for silicon microdosimetry.^33,34^ A silicon microdosimeter consisting of an array of microscopic p–n junctions based on the silicon-on-insulator technology has also been fabricated and tested with various hadron therapy radiation fields,^35–37^ including out-of-field dose-equivalent derivation in proton therapy.^38,39^

**Figure 2.**
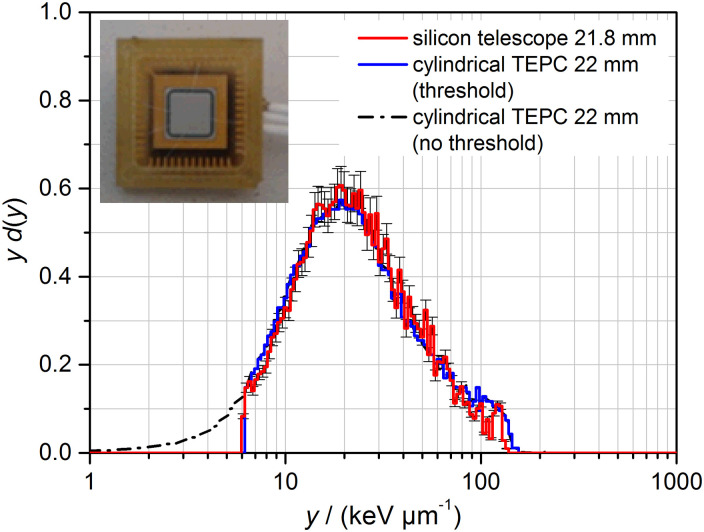
Microdosimetric spectra measured with a monolithic silicon telescope and a cylindrical tissue-equivalent proportional counter (TEPC) in a polymethyl methacrylate (PMMA) phantom at the distal edge of a clinical proton beam. Inset: a picture of the monolithic silicon telescope. Adapted from Agosteo et al^43^ with permission from Elsevier.

A complete and detailed characterization of hadron therapy beams (protons and carbon ions) was recently performed by Agosteo et al^40–44^ with a new device based on the monolithic silicon telescope technology. It consists of a microdosimetric diode device followed by a larger total absorbing device that is used to determine the total energy and type of the impinging particle. This structure optimizes the tissue-equivalence correction procedure so that the measured microdosimetric spectra are in good agreement with those acquired by mini-TEPCs (Figure 2).

**Figure 3.**
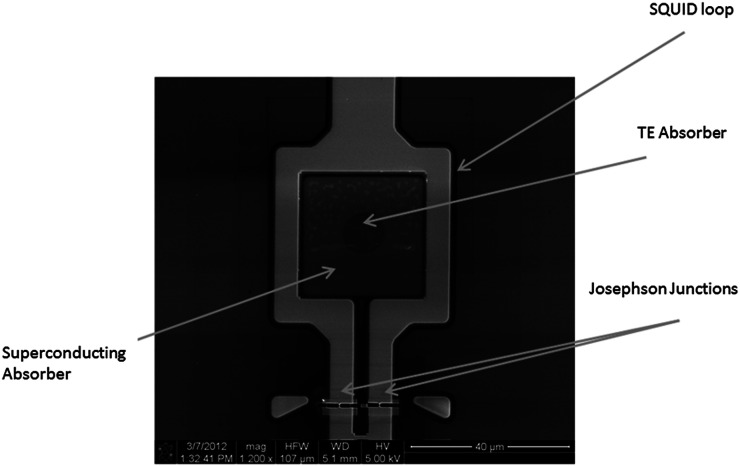
Scanning electron microscope image of an inductive superconductive transition edge sensor superconducting quantum interference device (SQUID)-based microcalorimeter.^47^ The entire field of view is about 100 × 100 μm. TE, tissue equivalent.

A novel detector for measuring the energy deposited at microscopic scales is currently under development at the National Physical Laboratory, Teddington, UK. The device is based on the inductive superconductive transition edge sensor (ISTED), which itself is based on a superconducting quantum interference device (SQUID) but contains the signal generating layer of superconductor within the SQUID loop.^45^ The device has been further modified for use in dosimetry by the inclusion of a tissue-equivalent layer on top of the superconducting layer as shown in Figure 3.^46,47^ During irradiation, energy deposited by the impinging radiation causes heating in the superconducting layer that in turn causes a change in the effective area of the superconducting absorber. This change is readily detectable by the SQUID loop and is measured as a change in voltage across the branches of the loop.

This type of detector has a number of advantages over conventional microdosimeters, such as it can be made geometrically similar to a cell or nucleus both in shape and size; it offers energy resolution down to approximately 0.2 eV; and it has a theoretical response time of less than a microsecond. The main drawback of this device is that it operates at temperatures <7 K, which increases the external dimensions of the device.

### Nanodosimetry

Nanodosimetry is concerned with measuring track structure down to nanometric resolution. At this scale, which is comparable to the dimension of DNA base pairs, the energy deposition is no longer the result of a large number of ionizations such that the *W*_gas_ value cannot be applied to obtain the imparted energy from the number of ionizations. Therefore, the characteristics of track structure are based on the formation of ionization clusters. The number of ionizations produced by a particular particle track in a specific target volume is a stochastic quantity called ionization cluster size. Track structure is characterized in nanodosimetry by the frequency distributions of ionization cluster size, or ionization cluster size distributions (ICSDs), rather than lineal energy distributions. In contrast to microdosimetry, the size of the target volumes considered in nanodosimetry is always smaller than the lateral extension of the penumbra of the primary particle track where interactions are owing to secondary electrons. Therefore, ICSD not only depends on the target size and geometry and the material composition of the target and its environment but also on the geometrical relation between the primary particle trajectory and the target. This is usually taken into account by specifying the smallest distance or impact parameter *d* of the primary particle trajectory and the centre of the target volume.

Relevant quantities in nanodosimetry are:

• *ν*: the ionization cluster size defined as the number of ionizations produced in the nanometric target volume by a single primary particle track, including ionizations produced in interactions of secondary electrons within the site
• 
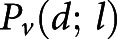
 or 
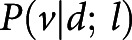
: the probability distribution of ionization cluster size, which depends on the impact parameter of the primary particle trajectory with respect to the target *d* and the size of the target *l*
• 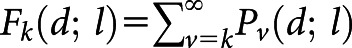: the (complementary) cumulative probability distribution of the ionization cluster size, giving the probability that an ionization cluster size of *k* or larger is produced in the target volume
• 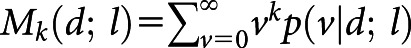: the *k*th statistical moments of 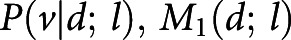 is also called the mean ionization cluster size.

Nanodosimetry is usually performed by filling a small volume with a low-density gas in order to simulate nanometric structures (DNA or similar), whereby the electrons or positive ions produced in single ionization interactions are measured. Some nanodosimeters are able to distinguish ionizations originating from the core or the penumbra of the track.

Similar to microdosimetry, nanodosimetry is based on a density scaling principle that allows equivalent ICSDs in target volumes of different sizes and material compositions to be obtained. Thus, two sites *A* and *B* are said to be equivalent when the mean ionization cluster sizes obtained in the two sites for the same radiation quality are equal, *i.e.* 
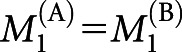
. It has been demonstrated that for targets that fulfil this equality, ICSDs obtained in propane are similar to those produced in nitrogen^48^ or in liquid water.^49–51^ The mean ionization cluster size *M*_1_ is related to the diameter of the sensitive volume and the mean free path for ionizing interactions of the primary particle with the medium *λ*_ion_, such that 
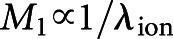
where the proportionality factor depends on the impact parameter *d*.

To date, three types of nanodosimeter devices (Jet Counter, StarTrack and Ion Counter) have been developed that are capable of measuring the frequency distribution of ionization cluster size in a gas. These nanodosimeters vary in the detected particle type, the operating gas and the size of the equivalent nanometric target in biological matter.

The Jet Counter at Narodowe Centrum Badán Jądrowych (NCBJ; Otwock-Swierk, Poland)^52^ detects positive ions produced by primary particles of electrons or ions in a jet of nitrogen gas propagating inside a cylindrical tube, where the number density of molecules can be adjusted to obtain biological target sizes in the range of 2–20 nm. The measured ICSDs relate to a central passage of the primary particle through the target. The Jet Counter is unique among the nanometric devices as it can be used to measure ICSDs of electrons.^53^ Recently, the Jet Counter has been used to measure nanodosimetric ICSDs for Auger electrons emitted by ^125^I.^54^

The StarTrack detector is installed at the Legnaro National Laboratories of the Italian Nuclear Research Institute (Padova, Italy). The target volume where interactions take place (*i.e.* the track detector) consists of an almost wall-less cylinder 3.7 mm in diameter and height defined by electrode wires. When operated at 3 mbar of pure propane gas, the mass per area of gas in this volume is about 2 µg cm^−2^, which corresponds to a length of 20 nm in materials of density 1 g cm^−3^.^55^ The target volume can be moved perpendicularly to the particle beam with an accuracy of 0.1 mm, enabling measurements for different impact parameters. Since the StarTrack device can detect electrons generated by an ion traversing the target volume or passing close by, it is able to distinguish ionizations generated in the core and the penumbra of the track, respectively. For each impact parameter, two sets of measurements are collected in order to filter background events and to obtain only the ICSDs generated inside the target volume.^56^

**Figure 4.**
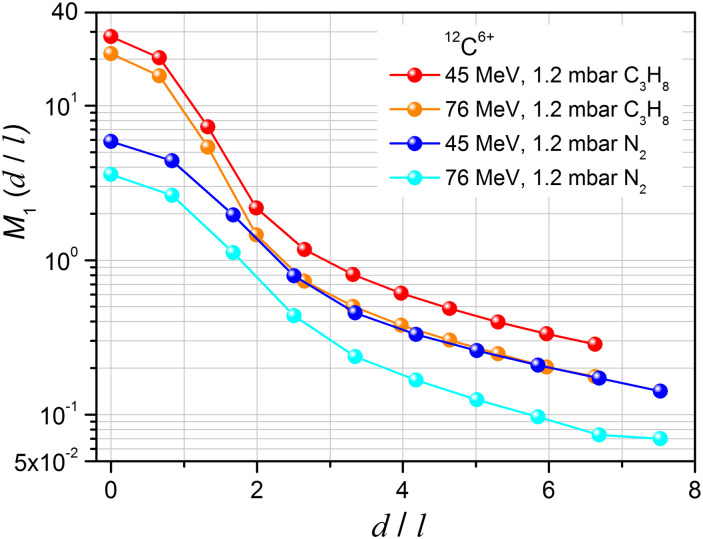
Mean ionization cluster size *M*_1_(*d*; *l*) for carbon ions of different energy for different impact parameters *d* given in multiples of the diameter *l* of the sensitive volume, measured with the Physikalisch-Technische Bundesanstalt (PTB; Braunschweig, Germany) ion counter in nitrogen and propane at 1.2 mbar pressure.

The Ion Counter at Physikalisch-Technische Bundesanstalt (PTB; Braunschweig, Germany)^57^ also detects positive ions produced in a wall-less gas volume. When operated with propane at 1.2 mbar, the Ion Counter simulates a volume element corresponding to a liquid water cylinder of about 3 nm in diameter. When operated with 1.2 mbar nitrogen, the diameter of the simulated volume reduces to about 0.5 nm in liquid water. While the PTB nanodosimeter has primarily been operated with nitrogen or propane gas, the possibility of using other operating gases, such as water vapour or gas mixtures of DNA ingredients, has recently been demonstrated. The Ion Counter is equipped with a position-sensitive trigger detector to record the position of the primary ion impinging on the detector surface. This position-sensitive detector enables the reconstruction of the primary particle's path, thus allowing the extraction of ICSDs with different impact parameters in order to discriminate between ICSDs originating from the core and the penumbra of the primary particle's track. Recently, a silicon microtelescope^58^ has been integrated into the Ion Counter to allow for simultaneous measurements of ICSDs and microdosimetric spectra. Figure 4 shows an example of measurements obtained with this device for the mean ionization cluster size 
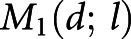
 produced by carbon ions of different energies and different impact parameters *d* (in multiples of the sensitive volume's diameter *l*) in 1.2 mbar of nitrogen and propane.

All three nanodosimeters benefit from computer simulations, particularly for checking the response of the detector in a well-known radiation field and irradiation geometry. The efficiency for extraction and detection of secondary particles, which are produced in the ionization process and detected by the respective nanodosimeters, are included in the simulation in order to reproduce the measured ionization frequency distributions.

A complete characterization of particle track structure requires the measurement of ICSD for all biologically relevant target sizes and impact parameters. As different target sizes may be relevant for different biological effects—such as tumour cell killing or inactivation, on the one hand, and normal tissue complications, on the other hand—one of the major challenges in nanodosimetry is the development of an instrument that is capable of measuring ICSD for targets covering a large range of simulated site sizes. This ambitious instrument development task would be simplified if particular ranges of relevant target sizes could be identified for which radiobiological effects correlate well with ICSD or parameters derived from them. Then, the envisaged multiscale nanodosimeter would have to only simulate the respective site sizes, and the remaining challenge would be to build a portable instrument for track structure measurements in different therapeutic beams.

### Track structure simulations

Differences in the spatial distribution of energy deposition at the subcellular scale are believed to be at the origin of the RBE of different radiation qualities.^18,19,59^ In this context, Monte Carlo codes are a well-adapted method for providing a realistic numerical simulation of these track structures on an event-by-event basis. These codes require physical models to enable the simulation of all the processes leading to an energy deposition or a change in the direction of the transported particle in the biological target. Monte Carlo modelling at the micrometre and nanometre level is challenging, both in terms of the physics modelling and code implementation. Not only are there theoretical challenges that have yet to be overcome^60,61^ but also the physical meaning of computed microdosimetric (or nanodosimetric) quantities need clarification. The growing need to perform simulations at such low energies and dimensions has led to the creation of new codes in the last few years and also to the improvement of several previously used codes.^62^ Another challenge relates to the simulation of biological tissue and its response, which is not straightforward, and there is no generic biophysical model to date. A well-performing Monte Carlo code would be one in which each track produced by the primary particle and by all of its secondaries can be followed in sufficient detail and in which the interactions are related to radiochemical interactions and afterwards to biological–chemical ones. This capability has yet to be achieved, although some codes can perform parts of such a task.^63^

For the simulation of microdosimetric spectra, it suffices to use the condensed history mode, which groups a large number of interactions in a single simulation step, given the large number of interactions along a track crossing the detector or site of interest. A summary of currently available codes and their characteristics with respect to the simulation of microdosimetric spectra is presented in Table 1.^65,67–75^

**Table 1.** Summary of the characteristics of some common Monte Carlo codes towards the calculation of microdosimetric spectra

Code name	Energy ranges/low kinetic cut-off	Microdosimetry applications	Low-energy physics models
MCNP6*^a^*^,69^	Photon γ (1 eV–100 GeV*^b^*) Electron e^−^ (10 eV–1 GeV) Positron e^+^ (10 eV–1 GeV) Proton p^+^ (1 MeV–1 TeV*^c^*^,^*^d^*) Heavy ions (5 MeV–1 TeV) Alpha particles α (4 MeV–1 TeV)	–MCNP6 has extended the minimum energy cut-off for photon and electron transport down to 1 and 10 eV, respectively –currently interested in adding molecular interaction cross sections for both photons and electrons	Five cards (LCA, LCB, LCC, LEA and LEB) control physics parameters of the following models: –Bertini –ISABEL –CEM03.03 –LAQGSM03.03 –INCL4 –ABL
MCNPX-2.70^70^	Photon γ (1 keV–100 GeV) Electron e^−^ (1 keV–1 GeV) Positron e^+^ (10 eV–1 GeV) Proton p^+^ (1 MeV–150 MeV*^d^*) Heavy ions (5 MeV–1 TeV) Alpha particles α (4 MeV–1 TeV)	–MCNPX was shown to be suitable when voxel dimensions of higher or equal to 1 μm are used to construct a voxelized phantom –it is inadequate for handling microdosimetric considerations required in cell culture irradiations because the uncertainties in measurements are large	Five cards (LCA, LCB, LCC, LEA and LEB) control physics parameters of the following models: –Bertini –ISABEL –CEM03 –INCL4 –FLUKA
PENELOPE*^e^*^,71^	Photo γ Electron e^−^ (100 eV–1 GeV) Positron e^+^	Some studies have been performed, but this code is not the first choice when working on simulations at the micrometric scale	–γ interaction models (Rayleigh and Compton scattering, photoelectric effect, electron–positron pair production) –e^−^, e^+^ interaction models (elastic collisions—MW model, inelastic collisions—GOS model, bremsstrahlung emission, positron annihilation)
GEANT4^72^	Low-energy packages*^f^* Default cut value: 1.0 mm*^g^*	GEANT4-DNA: adapt the general purpose Geant4 Monte Carlo toolkit for the simulation of interactions of radiation with biological systems at the cellular and DNA level (microdosimetry) –radiobiology, radiotherapy and hadrontherapy –prediction of DNA strand breaks from ionizing radiation –not limited to biological materials They are valid for liquid water medium only	–Livermore library (γ, e^−^) –Livermore library-based polarized processes –Penelope (v. 2008 as default) (γ, e^−^, e^+^) –ion parameterized energy loss GEANT-DNA–atomic de-excitation
	Livermore(γ, e^−^) (250 eV–100 GeV) Penelope (γ, e^−^, e^+^) (250 eV–1 GeV) Hadrons/ions up to 1 GeV GEANT4-DNA (4 eV–10 MeV)		
FLUKA*^h,^*^73^	Photon γ (1 keV–10,000 TeV) Electron e^−^ (70 keV–1000 TeV) Heavy ions (150 keV–1000 TeV) Charged hadrons [<10000 TeV/n] (100 keV–20 TeV)	FLUKA has been increasingly used for studies in microdosimetry: –applications in ion beam therapy treatment planning –simulations of microdosimetric quantities for carbon ions at therapeutic energies –investigations in the physical and biological effects of space radiation and developing mitigating strategies to reduce risk to humans	Neutrons: own cross-section library (P5 Legendre angular expansion, 260 neutron energy groups). Charged hadrons: combination of δ ray production with properly restricted ionization fluctuations (includes corrections for particle spin and electrons/positrons and “distant collision” straggling corrections) Electrons: complete multiple Coulomb scattering; cross sections of Seltzer and Berger; the Landau–Pomeranchuk–Migdal suppression effect. Photons: pair production; Compton effect with account for atomic bonds through use of inelastic Hartree–Fock form factors; photoelectric effect with actual photoelectron angular distribution; Rayleigh scattering
PARTRAC^74^	Photon γ (1 eV–100 GeV) Electron e^−^/positron e^+^ (10 eV–10 MeV) Proton p^+^ (1 keV–1 GeV) Heavy ions (1 MeV u^−1^–1 GeV u^−1^) Alpha particles α (1 keV–1 GeV)	Extended to electron, photon and ion interactions, DNA targets, double-helix and chromosome models, chromatin fibre in atomic resolution, liquid water cross sections, stochastic chemistry, track structures within heterogeneous targets, cross sections for ion interactions and simulations of radiation damage to DNA by ions and stochastic model of DNA DSB repair via non-homologous end-joining pathway	Photons (taken from EPDL97 data library): coherent scattering, photoelectric effect, Compton scattering, pair production, Auger electron and fluorescence photon emission, relaxation processes. Electrons: PWBA using models of dielectric response function of liquid water.^64^ Above 10 keV, the relativistic Bethe approximation is used, <500 eV a semi-empirical model. Protons and alpha particles: excitations, ionizations and charge changing processes of electron capture and loss <1 MeV semi-empirical models based on Rudd model are used. Heavy ions: PWBA and Bethe models
PTRAN^65^	Proton p^+^ (50–250 MeV)	PTRAN is a Monte Carlo code specific for the simulation of protons with energies of interest in proton radiation therapy. It has been widely used for treatment planning applications. Its application is restricted to the simulation of pencil beams in homogeneous water	Only protons of varying energy are tracked taking into account the following mechanisms: –energy loss in Coulomb collisions with atomic electrons is sampled from Vavilov energy straggling distribution using ICRU 49 stopping powers as average values –multiple scattering deflection due to elastic scattering by atoms is sampled from Molière distribution Energy losses in non-elastic nuclear interactions are based on fits to experimental data based on theorical considerations
TRAX^66^	It is limited to ions with energies less than a few hundred MeV u^−1^, electrons and (in the future) photons with less than a few MeV. The lower threshold (1–10 eV) is given by the available cross sections	TRAX uses single interaction Monte Carlo method to describe radiation action at the lowest possible level. Applications in nano- and microscale Study of nanolesions induced by heavy ions in human tissue Calculations on ion track structure and many related quantities, such as DNA DSBs and relative biological effectiveness Treatment plans in particle therapy	The purpose of TRAX is to properly describe creation and transport of low-energy electrons It has been extended to: –creation of Auger electrons –elastic ion scattering Ionization and excitation

DSB, double-strand break; GOS, generalized oscillation strength; MW, modified Wentzel.

^*a*^ Combinations of options for the physics models should be chosen with careful consideration. Although many combinations are allowed, inappropriate choices can lead to incorrect results.

^*b*^ If the source photon energy is set to 100 GeV, a bad trouble error results. This does not occur if the source energy is reduced to 99.9999 GeV.

^*c*^ While MCNP6 will allow particles up to 100 TeV in energy, only particle energies up to 1 TeV have been reviewed for accuracy.

^*d*^ The 1-MeV lower limit is a default cut-off; the MCNP codes can track protons down to 1 keV.

^*e*^ See the PENELOPE 2008 manual for code number for the various interaction events.

^*f*^ The low-energy packeges should not be used for modelling electromagnetic interactions of particles with a kinetic energy >1 MeV.

^*g*^ This threshold should be defined as a distance that is internally converted to energy for individual materials.

^*h*^ See FLUKA manual, http://www.fluka.org/content/manuals/FM.pdf.

For definition of acronyms and abbreviations for code names, subroutines, cards and models we refer to the code manuals.

If a nanometric description of the energy deposition in the target is needed, low-energy electrons (<1–10 keV) must be transported without condensed history approximations. However, for DNA constituents, there exists no complete set of electron cross sections, which are crucial for the calculated values of the mean free path, the type of interaction, energy loss and angle of emission of the particle. For this reason, most of the dedicated Monte Carlo codes for track structure simulations use only liquid water to describe the molecular composition of the biological target. An extensive review of these codes and the physical models used for describing the interactions of low-energy electrons or ions with the target has been presented by Nikjoo et al.^76^ Some quantum mechanical models have also been developed^77,78^ for describing inelastic interactions with molecules leading to energy deposition. However, most of the dedicated Monte Carlo codes include cross sections derived from the first Born approximation. In this theory, the representation of the target material is given by the dielectric response function of the medium. This function is based on the energy loss function, for which only very few experimental data exist,^79,80^ and they are obtained for liquid water in the optical limit (momentum transfer *q* = 0). Different theoretical models have been developed in order to extend the results for other values of the transferred momentum (*q* > 0).^81–84^ In particular, a dispersion model using Drude polynomials,^85,86^ which allows the calculation of cross sections for different ionization and excitation shells of liquid water, is used in many of the dedicated Monte Carlo codes for track structure simulation (PARTRAC;^74^ KURBUC,^87^ PITS04^88^ and Geant4-DNA^67^).

If a geometrical model for the DNA molecule (often studied in the frame of early biological damages assessment) is used as a target in the Monte Carlo simulation, the use of DNA-like material cross sections becomes important. In the framework of the BioQuaRT project, the first complete cross-section set of DNA constituents based on experimental data^89,90^ was developed for a simulation of electrons down to the ionization threshold.^91^ The DNA constituents of interest were tetrahydrofuran, trimethylphosphate and pyrimidine, serving as models for the deoxyribose, phosphate group and PY nucleobases, respectively. In these experiments, cross sections for total scattering were measured for energies between 6 and 1 keV, while differential elastic and double-differential inelastic scattering cross sections were measured for energies between 20 and 1 keV and scattering angles between 15° and 135°. To enable a convenient implementation in the simulation code, the cross-section data were interpolated by suitable model functions, offering an extrapolation of the measured differential cross sections to forward and backward scattering angles.

**Figure 5.**
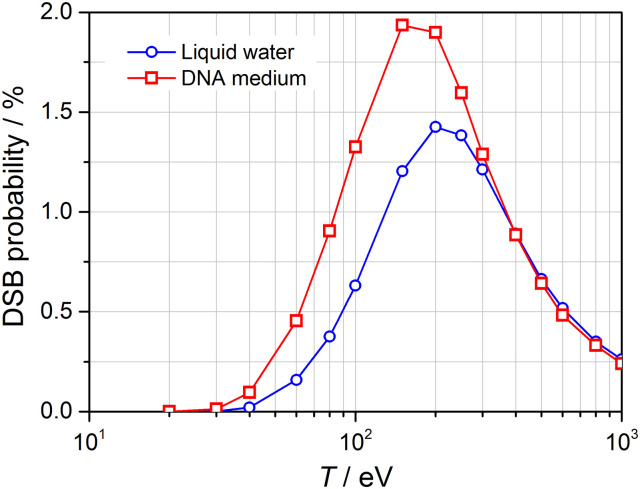
Probability of electrons with different energies *T* to produce a DNA double-strand break (DSB) when cross-section data of either liquid water or DNA medium are used in the simulations.^91^ The mass density was 1 g cm^−3^ in both cases.

A considerable difference of track structure parameters is observed when cross-section data of either liquid water or DNA medium are used in simulations. The consequence on predictions of the radiobiological effectiveness is shown in Figure 5, where estimated probabilities to obtain DNA double-strand breaks (DSBs) by electrons with energies <300 eV are significantly higher in DNA medium.

**Figure 6.**
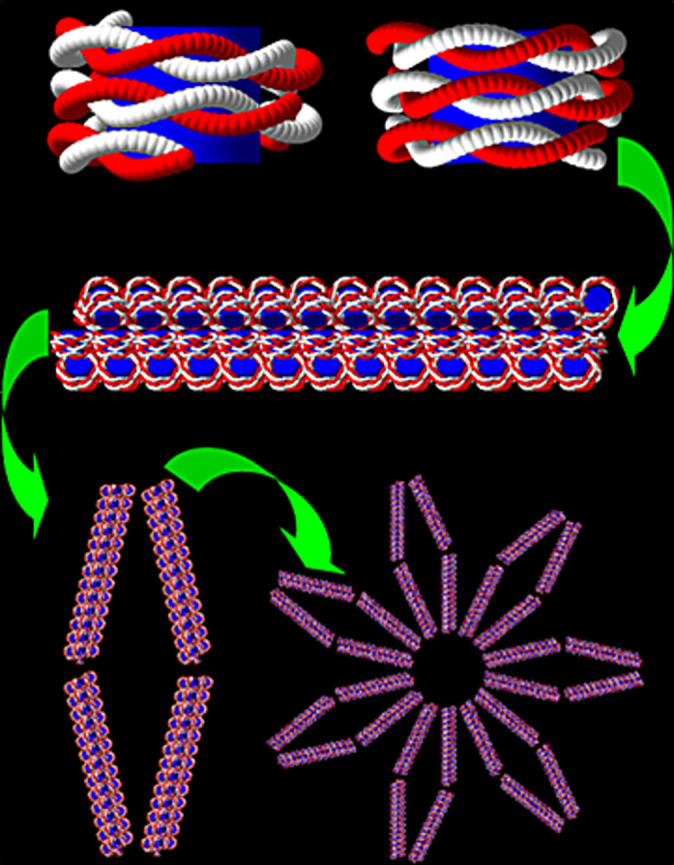
Different compaction levels of the DNA molecule implemented in the geometrical model used in the BioQuaRT project (data from Dos Santos et al^95^). The nucleosome shows the histone proteins (vertical cylinder) surrounded by two turns of the DNA double helix (backbone region and base pairs are distinct volumes within). This basic element is used for constructing chromatin fibres and fibre loops (simple and complex). The loops are then used to fill the chromosome territories in the cell nucleus (not shown).

In this case, the DSB probability was calculated from experimental ionization cluster size distributions using a combinatorial approach.^92^ Nevertheless, in most of the codes, the parameters needed for the calculation of the absolute number of DNA damages depend on the nanometric description of the DNA molecule target in the simulation. Different geometrical models have been implemented by different authors in the codes.^64,93,94^ In the BioQuaRT project, the geometrical model developed in the frame of the Geant4-DNA project^95^ is used in the multiscale simulation tool. The different compaction levels of the DNA molecule implemented in this model are shown in Figure 6.

This geometrical representation of the DNA molecule can be sufficient for calculating the direct DNA damages by considering the position of the energy transferred during the physical stage of interaction. Nevertheless, in order to take into account the “indirect effects” owing to the interaction with the radicals created by the irradiation in water surrounding the DNA molecule (chemical stage), a more realistic chemical description of the DNA is needed (for example see the RADAMOL code^96^). The geometry shown in Figure 6 is currently being adapted to be used in the BioQuaRT multiscale simulation tool, including the chemical stage developed in the frame of the Geant4-DNA project.^97^

### Quantification of radicals

As introduced in the last section, after energy deposition from the incident ionizing radiation, a range of radicals and reactive species are formed by the radiolysis of water molecules. Such radicals may interact directly on a short timescale (<10^−7^ s) with biomolecules, which are in close proximity to the interaction event or, after radical recombination and diffusion, react with key cellular component at some distance from the initial energy deposition. The damage caused to DNA through reactive radicals is classified as “indirect damage” (in contrast to the direct damage caused by the direct ionization of the biomolecules), and it is responsible for up to 70% of the total DNA lesions produced by radiation exposure to low-linear energy transfer (LET) radiation^98,99^ (for high-LET radiation direct action of ions is the major reason for their higher RBE^94^).

The majority of reactive species remain confined within 10–50 nm from the initial ionization,^100^ and this is of particular relevance in estimating radical recombination and the biological effectiveness of the indirect effect of radiation (*i.e.* radical induced) as clusters of DNA lesions represent more complex damage leading to more severe cellular consequences. It must also be noted that early biochemical modifications occurring during or shortly after radiation exposure might also cause oxidative stress, which continues to occur after the initial exposure. This is presumably owing to continuous endogenous production of reactive oxygen species (ROS) and reactive nitrogen species.^101^ Such oxidative changes might affect the irradiated cells as well as unirradiated neighbours and their progeny. There is now compelling evidence that the radicals produced by ionization of water (the hydroxyl radical, OH, in particular) play a key role in cell killing as well as sublethal cellular effects such as chromosomal aberrations.^102,103^

A vast amount of scientific literature exists on the chemistry in aqueous environments after radiolysis of water and the response of chemical systems to radiation. This has been investigated analytically using species-specific probes^104^ and scavengers to modify reaction pathways in a controlled manner and quantify the yield and spectrum of radicals produced. Direct observation of transient species using pulse radiolysis techniques has also provided information on reaction kinetics.^105^ For high-LET radiations, the contribution of radical species to DNA damage decreases despite the increased effectiveness per unit dose absorbed.^106^ This is attributed to a relative change in the yield of ROS owing to increased radical recombination favoured by their close proximity.^98^ Decreased yield of OH radicals has been reported for heavy-ion beams and linked to an increased production of molecular species such as hydrogen peroxide.^107^ Superoxide (O^−^_2_) has also been reported to increase with LET.^108,109^

Species-specific chemical probes can be used to quantify the relative concentrations of different biologically significant radiolysis products as a function of LET. Imaging of these probes in solid matrices or cells is also possible and can provide information on the spatial distribution of reaction events. Generally, fluorescent probes offer higher sensitivity and can be detected using a variety of analytical techniques. These include optical microscopy, which makes the probes particularly attractive for detection and quantification of reactive radical species. Numerous fluorescent probes have been suggested and used in both cell cultures and bulk solutions.^104,110^ Table 2 reports key specification of various probes.

**Table 2.** List and characteristics of the most employed probes in quantification of reactive radical production

Probe	ROS detected	Excitation/emission wavelength (nm)	Fluorescence product	Specificity	Notes
Hydroethidine	O_2_^−^	520/610	Ethidium (E)	Can also be oxidized by a variety of ROS (including H_2_O_2_) and reduced by cythochrome c	Interference problems due to fluorescence from other catalysis products
2-chloro-1,3-dibenzothiazolinecyclohexene (DBZTC)	O_2_^−^	485/559	DBZTC-oxide (DBO)	500:1 O_2_^−^:H_2_O_2_ reactivity	Long reaction kinetics (10 min) Narrow pH range (7.2–8.2) Could be used in combination with DCF probes (H_2_O_2_)
Dichlorodihydrofluorescein (DCFH)	H_2_O_2_	498/522	2, 7-dichlorofluorescein (DCF)	Can be oxidized by other peroxides Relative insensitive to O_2_^−^ Low reactivity with OH	2-electron process Require catalyst (metal/HRP) HRP alone can oxidize DCFH Photo-reduction in visible light and ultraviolet A
Amplex Red (AR)	H_2_O_2_	563/587	Resorufin	It requires HRP Can also be used for O_2_^−^ with superoxide dismutase which converts O_2_^−^ into H_2_O_2_ Interference from substances that can oxide HRP	Low background (spectra Em/Ab with low interference) High fluorescence power, high stability Further oxidation of resorufin (at very high H_2_O_2_ concentrations) might cause decreased sensitivity Very pH sensitive. Not stable at pH >8.3
Coumarin-3-carboxylic acid (3-CCA)	OH	350, 395/450	7-OHCCA	Very specific for OH	Highly sensitive to pH Well characterized (dose, LET, pH, time, …) Dose rate effect with no pure compound

HRP, horseradish peroxidase; ROS, reactive oxygen species.

Coumarin-3-carboxylic acid is a non-fluorescent organic chemical compound (C_10_H_6_O_4_) that upon interaction with hydroxyl radical converts to 7-hydroxyl coumarin-3-carboxylic acid (among other products), which is a highly fluorescent substance (excitation wavelength peaks at 395 and 350 nm and emission at 450 nm). Only 4.4% of the radiation-produced OH is detected through the production of 7-hydroxyl coumarin-3-carboxylic acid with a reported *G*-value of 0.123 molecules per 100 eV in low-LET radiation.^111^ The interaction of coumarin with the hydroxyl radicals is a single-step process that does not require additional catalysts. The intensity of the fluorescent-irradiated solution is proportional to the number of hydroxyl coumarin molecules, which is related to the yield of OH produced, and therefore the amount of dose absorbed. Coumarin has been suggested as a dosimeter in both bulk solutions and biological samples (low toxicity) owing to its linear dose response over a wide range, good reproducibility (±2%) and great stability over time (±3%).^111–113^ Dose linearity response has also been confirmed for a range of LET radiations (0.5–2000.0 keV µm^−1^).^114^ Another promising fluorescent system is hydroethidine, which can be used to detect superoxide radicals in bulk materials and in cells, although care needs to be taken in the interpretation of fluorescence owing to interference from other oxidative pathways.^115^ The excitation and emission wavelengths used are around 510 and 590 nm, respectively.

### Reference radiobiological data

In current radiobiology practice, biological effects imparted by ionizing radiation are linked to the classic unit absorbed dose. The interest in the spatial distribution of energy deposition into cells or cell nucleuses from a microdosimetric point of view and its relation to induced DNA damage began around three decades ago.^18,116–120^ An aspect of this relation is the correlation of the physical interaction at the level of one particle track in one cell to the initial creation of cellular damage. Experimental evidence points to DNA as one of the key targets (direct or indirect) of ionizing radiation.^121,122^ Among the different types of DNA damages, DNA DSBs are thought to be at the root of chromosome aberrations, mutation induction and cell death.^123,124^ Among the different techniques for measuring DSBs, one of the initial and most robust is pulsed field gel electrophoresis. It allows resolving and measuring of DNA fragments, which originate from the insertion of breakage points in the filaments of chromatin that constitutes the chromosomes (from 0.1 kbp and up to 10 Mbp).^125^ The fraction of DNA fragments of this size is correlated with the number of radiation-induced DSBs. However, to generate such fragments, it is necessary to expose the cell nuclei to very high absorbed doses (in the order of 100 Gy),^126^ complicating the study of damage at the scale of single particle tracks.

Recent advances in irradiation techniques and molecular biology have enabled the observation and quantification of DNA damage of individual cells to single particles of ionizing radiation, rather than averaging the effect over multiple cells. These advances have been accomplished with single-ion microbeams that offer the possibility to deliver one particle in a specific area of the cell, nucleus or cytoplasm, with a micrometric spatial resolution.^127–129^ In parallel, progress in cell imaging has improved the resolving power, which is defined as the minimum distance between two objects at which they can be distinguished separately. Recently, the resolving power has been improved from around 100 nm^130^ to tens of nanometres using stochastic optical reconstruction microscopy.^131^

**Figure 7.**
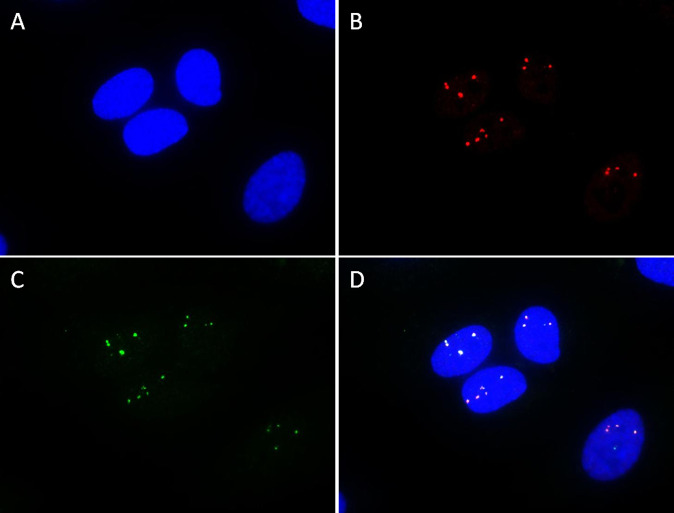
Representative images of 53BP1 and γ-H2AX foci in primary human umbilical vein endothelial cells after irradiation to exactly five alpha particles (20 MeV, 37 keV μm^−1^) per cell at the PTB microbeam. (a) Nucleus stained with 4′,6-diamidino-2-phenylindole (DAPI), (b) 53BP1 immunodetection. (c) γ-H2AX immunodetection and (d) merged.

The observation of foci formation (*e.g.* phosphorylation of H2AX on ser139 or the recruitment of 53BP1) at the sites of DNA DSBs by immunofluorescence (specific antibody tagged with fluorochrome) (Figure 7) and the analysis of their characteristics (location, size, shape, fluorescence intensity) makes a foci-based assay well suited to study the damage produced along the track of a particle at a low fluence such as one particle per cell.^132,133^ This type of biological measurement can be adapted to the establishment of reference radiobiological data to study biological effects at the submicrometre scale. These reference biological data, combined with detailed information on particle track structure, will help the foundation of new dosimetric quantities.

While the number of initial DNA damages inflicted by ionizing radiation of different qualities is relatively invariant per unit dose, the fraction of these damages that can lead to lethal events can vary significantly across the spectrum of ionization densities along particle tracks. In fact, this very feature lies at the heart of the increased effectiveness at the same radiation dose of particle therapy compared with photon therapy. Experimental measurements of initial DNA damage, which evaluate “late” biological damage from a set of experimental assays measuring the persistence or “fixation” of early damage induced by radiation, can benefit the comprehension of biologically relevant dosimetry. From this perspective, the classical clonogenic survival assay can be complemented by reference assays that study the genomic integrity of single cells, such as the cytokinesis-block micronucleus assay^134^ and other assays looking at a variety of forms of chromosome damage, as well as mutation induction assays. Experimental results from these techniques require a concerted effort to obtain new dosimetric quantities where the roles of physics and of biology are clearly distinguished. This is the approach used in the BioQuaRT project, within the framework of its multiscale model that will be introduced below.

Currently, precise irradiations are carried out at a microbeam facility,^129^ which provides high-LET *α*-particles and low-LET protons with energies of 3–20 MeV. This range of ions and energies allows the selection of radiation qualities with LET values between 3 and 200 keV µm^−1^, which covers almost entirely the range from diagnostic X-rays to naturally occurring *α*-particle radiation. It also provides *α*-particles with an ionization density comparable with the average LET in the spread-out Bragg peak encountered in radiotherapy with carbon-12 (^12^C) ion beams (in the order of 100 keV μm^−1^). As it is well known that beams of different ions having the same LET may have a different biological effectiveness owing to their different track structure,^126,135–138^ microbeam irradiation studies with ^12^C ions will be needed in the future to quantify the different response of the biological system to *α*-particles and ^12^C ions with this LET.

### Clinical relevance/need

In addition to the different depth dose curves of particle beams compared with X-rays, the clinical application of these beams has also to account for differences in the biological response at the same dose, which is typically quantified by the “RBE”. For protons, a constant RBE of 1.1 is generally assumed for patient treatments,^139^ although it is known that the RBE is not constant across the irradiated volume. Using a monolithic silicon telescope in conjunction with the RBE data from cell survival studies, it has been demonstrated, for example, that RBE of a proton therapy beam increases dramatically towards the end of the spread-out Bragg peak.^140^ There is increasing research interest to consider RBE variations (depending on the local energy spectrum and tissue type) in treatment planning for protons. For carbon ions, however, the RBE is in the order of 2–5, and it is mandatory to account for local variations in clinical treatment planning,^141,142^ which are based on biophysical model estimations.^143,144^ A major drawback is that these RBE models are mainly based on data from cell culture measurements *in vitro*, and it is very challenging to validate these models in clinical studies. Furthermore, different centres often use their individual approaches, hence it is difficult to compare treatment plans or clinical experience between different carbon ion facilities.^145^ Therefore, the reporting of “RBE-weighted absorbed dose” [in units of Gy (RBE)],^146,147^ previously given in units of cobalt grey equivalent or GE, is not sufficient, and there is a need for standardization and more objective measurements of local radiation quality. This will go beyond individual approaches for “biological dosimetry” with cell culture assays^148^ and would facilitate the integration of the complex biological response into clinical treatment planning systems. In the long run, novel quantities to describe the local radiation quality on a macroscopic level (typical voxel size of 1 mm^3^ in a three-dimensional patient geometry) may be used for treatment plan optimization, evaluation and dosimetric verification. These quantities will presumably be a function of both microdosimetric and nanodosimetric distributions and may allow for new radiotherapy planning strategies aiming for homogeneous radiation quality and hence biological response across the target volume, provided this response is constant for a given biological system if the radiation quality as represented by these quantities is constant. A pre-requisite to this is the availability of routine measurement tools, preferably integrating microdosimetric and nanodosimetric devices in a single set-up, with high spatial resolution. Potential candidate technologies to form the basis of such devices include the silicon microtelescopes and microcalorimeters discussed above.

## FUTURE OUTLOOK—NEW STANDARDIZED BIOLOGICALLY RELEVANT DOSIMETRIC QUANTITIES

Given the complexity of the initiation and occurrence of biological processes on various scales that depend on both ionization and non-ionization events, a multiscale approach is needed to lay the foundation for new physical quantities relating track structure to RBE in proton and ion beam therapy. The BioQuaRT project^15^ aims to explore this approach by developing measurement and simulation techniques for determining the physical properties of ionizing particle track structure on different length scales from 2 nm (diameter of DNA double helix) to 10 µm (diameter of cell nucleus), which would allow a multiscale characterization of the radiation qualities used in ion beam therapy. The following objectives are set out in BioQuaRT to realize its overall aim:

• Microcalorimeters will be developed for the direct measurement of lineal energy^46,47^ (radiation quality) and will be compared with state-of-the-art conventional microdosimeters that measure ionization (mini-TEPC^29,30^ and Si-microtelescopes^41,42^) with the eventual goal of assessing whether corrections are needed for those conventional microdosimeters.
• Measurement techniques for particle track structure at different length scales down to the nanometre range will be further developed allowing a multiscale characterization of radiation qualities. A comparison of the Jet Counter,^52^ StarTrack detector^55^ and Ion Counter^57^ will be made following modifications to improve their performance as well as further development of the simulation tools used in their data analysis.
• A prototype system will be developed to determine the spatial distribution of biologically relevant reactive species and the relative yield of their production as part of the physical characterization of the pathway of indirect radiation effects on cellular operation.
• A comprehensive multiscale simulation tool, which will include data for radiation interaction cross sections with DNA and the production rates of radical species, will be developed relating the characteristics of track structure to the biological consequences of radiation interaction.
• Biological reference data and benchmarks for the multiscale model will be created by performing radiobiological assays in cultured tissue cells to quantify the induction of initial DNA damage as well as late effects such as the misrepair of DSBs.

Most of this exploratory work will be performed using single-ion microbeams of protons and α particles (and possibly ^12^C-ions) and will be extended later to clinical beams. The experimental physical characterization of track structure comprises microdosimetric spectra and the measurement of ionization cluster size distributions at the nanometre scale. This allows establishing the link between energy depositions at the cell or cell nucleus scale and energy depositions that are more directly related to DNA damage. The combination of both microdosimetric and nanodosimetric characteristics of track structure can subsequently be used to overcome the non-uniqueness in the relation between mean values of microdosimetric spectra (such as 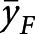) and biological effects.

Since the multiscale simulation tool is a rather novel concept, it is discussed here in further detail. Its aim is to include improvements in the understanding and quantification of the different stages of radiation action leading to primary damage at the cellular level, starting from the physical energy deposition and considering all known pathways to cellular damage. In general, a multiscale simulation model should enable a biologically relevant weighting of physical quantities at different length scales in a useful and practical way with the purpose of defining a biologically more relevant quantity for hadron therapy. Such a multiscale model was presented by Solov'yov et al.^149^ While the analytical approximations and hypothesis included in that proposal probably result in too crude an approach, one must realize that, at present, available computing resources do not allow simulating the whole chain of events in full detail and analytical approaches for some of the steps may prove essential for a practical and efficient implementation.

The multiscale simulation tool under development in BioQuaRT will be primarily based on the Monte Carlo calculation of ionization and energy deposition at the nanometre scale. At this first simulation stage, it is important to strive for the highest achievable accuracy since these nanometric distributions are at the origin of the biological consequences and differences at this scale are responsible for the variation in the biological effectiveness between different radiation qualities. Specifically, this first simulation stage requires the development of the physical models for radiation interaction cross sections with the biomolecules in the target and experimental data for evaluating these physical models, as these cross-section models define the outcome of the Monte Carlo simulation. This stage also requires the geometrical target description that determines the energy transfer points from which chemical bond breaks may originate. The multiscale tool will further include a simulation of the chemical stage, which follows the creation of radicals by the ionizing radiation interactions in the cells and gives rise to indirect effects, as this is also fundamental to obtain a complete evaluation of the biological damage. In this respect, the quantification of radicals in the BioQuaRT project and the study concerning their contribution to DNA damage are of great importance for the validating the chemical part of the multiscale simulation tool.

By comparison of the multiscale model with the experimentally determined physical characteristics of track structure and the corresponding outcome for the aforementioned biological end points for ion beam radiation of different energy, the relevant quantities can be identified that need to be simulated in order to quantify the biological quality of the macroscopic irradiation. The input parameters needed in the multiscale simulation tool are the conventional physical characteristics of the radiation field (*i.e.* the spectrum of particle types and their fluence and energy distribution), which preserve the reference of the multiscale model results to quantities currently used in radiotherapy. Moreover, owing to the fact that the Monte Carlo simulation in the multiscale model is based on an open-source approach, the user can take advantage of the possibility of using other physical models available. For example, simulating the passage of the primary beam through different elements in the beam line before the interaction with the biological target, the results obtained in the multiscale model will take into account the radiation field impinging on the target.

**Figure 8.**
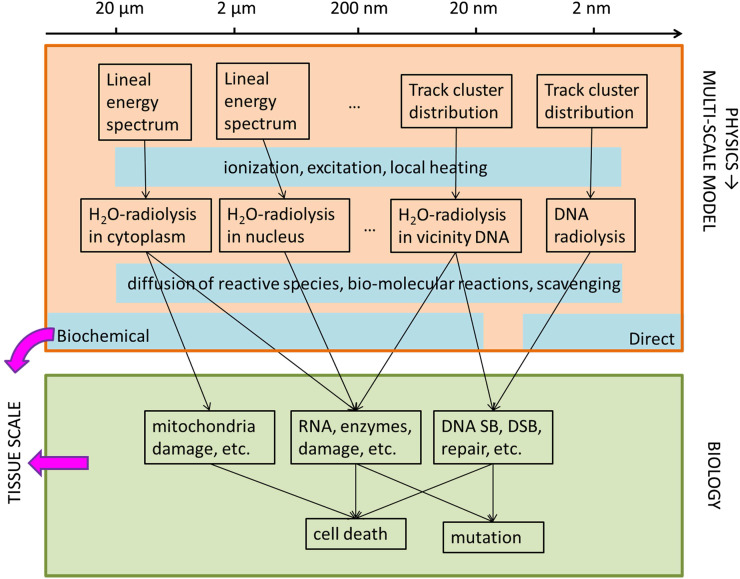
Schematic view of a possible multiscale model. The large upper box represents the physics part of the process. The dots indicate a continuum of physical parameters and radiochemical process at different length scales. The thick arrows indicate that there are other scale levels of importance that are not yet considered in the proposed multiscale model. The large lower box represents various biological effects. DSB, double-strand break; SB, strand break.

A top level schematic representation of a possible multiscale model is shown in Figure 8. This figure does not necessarily present a complete model but shows, nevertheless, that it is possible to enumerate all relevant processes. On the other hand, it also illustrates that the complexity, even on the pure physics side with a limited number of physicochemical end points, is so high that the required computing power to model all these processes in detail is likely inaccessible, making approximations unavoidable. The implication of such approximations on the uncertainty of a model is difficult to assess and maybe even more difficult to quantify given the uncertainties on biological data. Appropriate methods to quantify such uncertainties are being investigated.^150^

Although it is still far too early to specify how eventually a sensible weighting of physical quantities at the micro- and nanoscale will have to be carried out, a tentative generic way of expressing this weighting could be as follows:
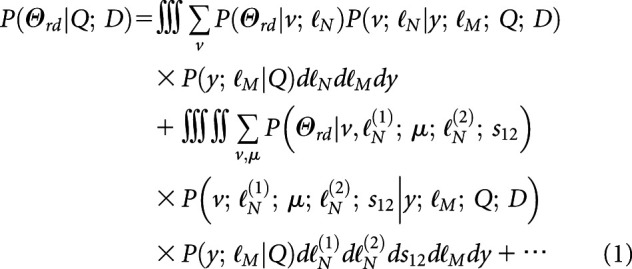


The left-hand side is the conditional probability that irradiation of a biological system with radiation of quality (*Q*) to a macroscopic absorbed dose (*D*) will lead at the end of the physicochemical stage to a particular initial radiation damage pattern *Θ*_rd_. This could, for instance, be a certain spatial distribution of complex DNA DSBs within a cell nucleus. How to best quantify this initial radiation damage pattern is a task still to be solved.

This quantity given in Equation (1) would then be used in biological models for predicting biological outcome of irradiation which generically can be written as:
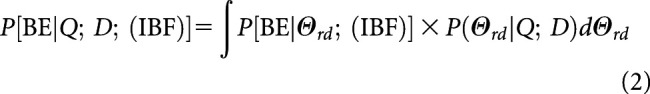
where 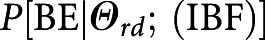 is a biological weighting function representing the conditional probability that for the particular biological system, the biological end point under consideration is the result of the (physical or physicochemical) radiation damage pattern 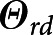. In this equation, (IBF) stands for the collection of all purely biological factors that influence the biological outcome, such as the fractionation scheme or difference in repair capacity of cells from different individuals.

In Equation (1), the triple dots indicate potential higher order terms, while the other quantities have the following meaning: 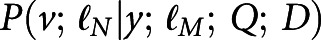
is the conditional probability that an ionization cluster of size is produced in a target volume of size 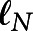, if a value of lineal energy *y* is obtained in a microscopic site of size 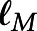. Analogously, 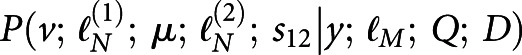 is the conditional probability that ionization clusters of size *v* and *μ* are produced in target volumes of size 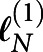 and 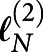, respectively, that are separated by *s*_12_, if a value of lineal energy (*y*) is obtained in a microscopic site of size, 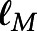.
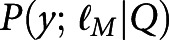
 is the probability of having a lineal energy *y* in a microscopic site of size 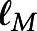 and can be obtained from conventional microdosimetric measurements. The aforementioned two conditional probabilities 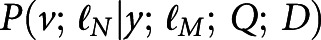 and 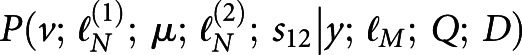 are also, in principle, measureable with a nanodosimeter of multiscale measurement capabilities that still needs to be developed in the future. However, measurements within the BioQuaRT project using the existing nanodosimeters will determine these distributions for single ion tracks and a range of target sizes, and the multiscale simulation tool under development will allow for an extrapolation to other target sizes and for studying the correlations between ionization clusters contained in the second aforementioned distribution.


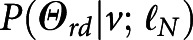
 would be one of the physical weighting functions and gives the conditional probability that an ionization cluster of size *v* in a target volume of size 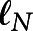 will lead to the radiation damage pattern 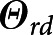 at the end of the physicochemical stage of the radiation interaction. The second weighting function 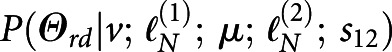 is the conditional probability that the radiation damage pattern 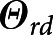 occurs given that ionization clusters of sizes *v* and *μ* are produced in targets of size 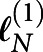 and 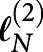, respectively, which are at a distance *s*_12_ apart from each other. In their dependence on the target volume size, both weighting functions would be expected to have pronounced maxima in the vicinity of values representative of sensitive biological targets, such as the diameter of the DNA double helix, the thickness of the cell nucleus membrane, the size of a histone and of the sizes of important organelles such as the mytochondria. Hence, with respect to these independent variables, the integrals may simply reduce to sums over a few relevant target sizes. The dependence on the separation, on the other hand, would presumably be a smoothly varying function. One important purpose of the multiscale simulation tool under development is to provide a means for obtaining data from which the functional relations entering the weighting functions can be determined.

The practical implementation of the multiscale model will require the measurement of physical track structure data including microdosimetric spectra and ionization cluster size distributions at the nanometre scale for therapeutic beams. To make the experimental determination of those quantities a viable option in clinical practice, easy-to-operate devices need to be developed. While the silicon microtelescope devices described above are an example of a significant step forward in realizing this for microdosimetric quantities, for measurements at the nanoscale, this remains at present far from obvious how this can be achieved.

## CONCLUSION

This review on the state-of-the-art microdosimetry, nanodosimetry, track structure simulations, quantification of reactive species, reference radiobiological data, cross-section data and multiscale models of biological response has been presented in the context of defining new physical quantities that enable a transparent separation of the physical and biological processes concerning biological effects of ionizing radiation. The benefit of this separation with respect to biological effects, such as, for instance, fractionation, would be that different beams having an identical physical beam quality according to such a new quantity, would behave exactly the same as a function of fractionation. The need for a clear definition of the quantities to be measured (in terms of quantifying interactions but also in terms of the relevant medium) is highlighted. The present status of efforts to achieve direct lineal energy measurements, rather than deriving lineal energy from ionization measurements, is discussed as well as efforts to improve the measurement and simulation of nanodosimetric quantities, quantification of reactive chemical species and progress to improved biological data by using a rigorous metrological approach. A conceptual description of a multiscale model that can form the basis of the new quantities is described, and it is anticipated that the European metrology project BioQuaRT will be able to contribute substantially to the progress towards realizing such a multiscale modelling tool. A generic formal expression of how the weighting of physical quantities at different length scales and the separation from biological effects could possibly be carried out is presented for further discussion, debate and refinement.

## FUNDING

The work that led to this review is supported by the European Metrology Research Programme (EMRP) joint research project BioQuaRT, which has received funding from the European Union on the basis of decision number 912/2009/EC. The EMRP is jointly funded by the EMRP participating countries within EURAMET and the European Union.
